# Next generation sequencing analysis reveals a relationship between rDNA unit diversity and locus number in *Nicotiana* diploids

**DOI:** 10.1186/1471-2164-13-722

**Published:** 2012-12-23

**Authors:** Roman Matyášek, Simon Renny-Byfield, Jaroslav Fulneček, Jiří Macas, Marie-Angele Grandbastien, Richard Nichols, Andrew Leitch, Aleš Kovařík

**Affiliations:** 1 Institute of Biophysics, Academy of Sciences of the Czech Republic, v.v.i, Královopolská 135, Brno, CZ-612 65, Czech Republic; 2 Queen Mary University of London, School of Biological and Chemical Sciences, Mile End Road, London, E1 4NS, UK; 3 Department of Ecology, Evolution and Organismal Biology, Iowa State University, Ames, IA 50011, USA; 4 Biology Centre, Academy of Sciences of the Czech Republic, Institute of Plant Molecular Biology, Branišovská 31, České Budějovice, CZ-370 05, Czech Republic; 5 Institut Jean-Pierre Bourgin, Laboratoire de Biologie Cellulaire, INRA-Centre de Versailles, Versailles Cedex, F-780 26, France

**Keywords:** rRNA genes, Concerted evolution, Plants, Chromosomes, DNA repeats homogenisation

## Abstract

**Background:**

Tandemly arranged nuclear ribosomal DNA (rDNA), encoding 18S, 5.8S and 26S ribosomal RNA (rRNA), exhibit concerted evolution, a pattern thought to result from the homogenisation of rDNA arrays. However rDNA homogeneity at the single nucleotide polymorphism (SNP) level has not been detailed in organisms with more than a few hundred copies of the rDNA unit. Here we study rDNA complexity in species with arrays consisting of thousands of units.

**Methods:**

We examined homogeneity of genic (18S) and non-coding internally transcribed spacer (ITS1) regions of rDNA using Roche 454 and/or Illumina platforms in four angiosperm species, *Nicotiana sylvestris*, *N. tomentosiformis*, *N. otophora* and *N. kawakamii*. We compared the data with Southern blot hybridisation revealing the structure of intergenic spacer (IGS) sequences and with the number and distribution of rDNA loci.

**Results and Conclusions:**

In all four species the intragenomic homogeneity of the 18S gene was high; a single ribotype makes up over 90% of the genes. However greater variation was observed in the ITS1 region, particularly in species with two or more rDNA loci, where >55% of rDNA units were a single ribotype, with the second most abundant variant accounted for >18% of units. IGS heterogeneity was high in all species. The increased number of ribotypes in ITS1 compared with 18S sequences may reflect rounds of incomplete homogenisation with strong selection for functional genic regions and relaxed selection on ITS1 variants. The relationship between the number of ITS1 ribotypes and the number of rDNA loci leads us to propose that rDNA evolution and complexity is influenced by locus number and/or amplification of orphaned rDNA units at new chromosomal locations.

## Background

In most eukaryotes, 5S and 18–5.8–26S nuclear ribosomal DNA (rDNA) units occur in tandem array at one or several loci. Each large rDNA unit (designated 45S and 35S for animals and plants, respectively) contains three different rRNA genes (18S, 5.8S and 26S), which are separated by two internal transcribed spacer sequences (ITS1 and ITS2). Each group is separated by the intergenic spacer (IGS, [1]). The genic regions are highly conserved, whereas ITS divergence is sufficient to resolve species relationships within most genera. The IGS, which contains the transcription-start site and genetic and epigenetic features that influence the regulation of downstream genes, diverges more rapidly than the ITS.

Of particular interest to evolutionary biologists is the pattern of divergence of the whole rDNA array known as concerted evolution, in which the units of the rDNA array are very similar within a species but diverge between species. This pattern suggests that the arrays are subject to homogenisation – so that novel variants arising by mutation spread relatively rapidly along the array within any one species [2-4]. Computer modelling has suggested homogenisation would act to reduce mutational load and could therefore be favoured by selection [5,6]. An alternative model of multigene family evolution, perhaps most generally applicable to small multigene families, argues for birth and death of duplicate gene copies associated with selection, explaining relatively high diversity observed in gene families [7,8]. It has been suggested that this model might explain the unusually high levels of ITS and IGS polymorphisms in some species (up to 40% in some cases) [9-11].

In angiosperms, rDNA evolution has been challenging to study, since there are many copies of the large 35S unit, amounting to thousands, or tens of thousands of units [12] distributed over one or several chromosomal loci [13]. High copy-number and sequence conservation of tandemly-arranged units make the analysis of the structure and organisation of the entire array difficult by classical cloning and sequencing approaches. Consequently, rDNA homogeneity at the single nucleotide polymorphism (SNP) level has not been comprehensively studied in any organism carrying more than a few hundred copies of the 35S rDNA unit. The advent of next generation sequencing (NGS) allows such an analysis for the first time [14,15]; in this study we therefore took advantage of NGS to explore rDNA homogeneity in four angiosperm species in the genus *Nicotiana* (Solanaceae).

*Nicotiana* is an ideal choice for the study of rDNA variation because we can take advantage of existing studies on the evolutionary processes operating across the genus. These studies at the cytogenetic [16-18], molecular [19,20] and genomic [21-23] levels have given insights into species relationships and patterns of species divergence. There are several phylogenetic studies examining patterns of sequence divergence across the genus [24-27], one of which used ITS sequences to infer species relationships [27]. These data revealed the presence of allopolyploids and complex patterns of interspecific hybridisation at the diploid level. Cytogenetic studies in *Nicotiana* have revealed that 35S rDNA can occupy as many as five loci on different chromosomes [17,28,29] and in some species of section *Tomentosae* sub-regions of the rDNA array may also be dispersed [30]. Rapid amplification of novel 35S rDNA units has been observed in the fourth generation descendents of synthetic allopolyploids, artificially created to resemble natural *N. tabacum*[31]. Indeed the natural allotetraploid *N. tabacum*, thought to have formed <200 000 years ago from relatives of modern *N. sylvestris* and *N. tomentosiformis*[32,33], shows signatures of rapid concerted evolution, where most of the progenitor 35S rDNA units have been replaced by a variant that most closely resembles the *N. tomentosiformis* unit type [34,35].

**Figure 1:**
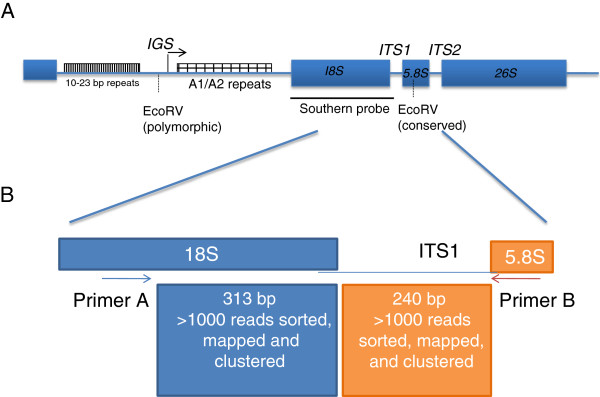
**(A) Structure of 35S rDNA unit in *Nicotiana *(redrawn from**[34]**).** The transcription start site is arrowed. IGS = intergenic spacer, ITS = internal transcribed spacer (B) PCR primer binding sites to the 18S and 5.8S rDNA unit and 454 sequencing reads generated. The total amplicon length was 650 bp.

In this work, we addressed inter- and intragenomic homogeneity of rDNA arrays in four diploid *Nicotiana* species using two platforms of next generation sequencing (Roche 454 and Illumina) coupled with classical Sanger method. We sequenced amplicons covering equal portions of coding (18S gene) and non-coding (ITS1) regions (Figure 1). With these data, we compared the families and levels of divergence of units in both regions and interpreted the data in relation to the numbers of rDNA loci and levels of divergence in the intergenic spacer sequences (IGS). We found evidence for near-complete homogeneity of coding sequence irrespective of locus and copy-number. The non-coding region shows significantly higher divergence within those species that harbour multiple rDNA loci.

## Methods

### Plant material

The following accessions were used: i) *Nicotiana sylvestris* Speg. & Comes ac. ITB626 originating from the Tobacco Institute, Imperial Tobacco Group, Bergerac, France. ii) *Nicotiana tomentosiformis* Goodsp. ac. NIC 479/84 (Institute of Plant Genetics and Crop Plant Research, Gatersleben, Germany). iii) *Nicotiana otophora* Griesebach ac. 406/76 (Royal Botanic Gardens, Kew, UK) and iv) *N. kawakamii* Y*.* Ohashi (voucher FN568429, Natural History Museum, London, UK).

### Preparation of sequencing amplicons by emulsion PCR

Emulsion PCR was used in all steps in order to prevent formation of chimeric DNA during amplification. We first separated individual rDNA units in genomic DNA by *Hin*dIII restriction enzyme digestion, for which there is at least one restriction site distal to the 18S-5.8S region [34]. About 15 μg of genomic DNAs from *N. sylvestris, N. tomentosiformis, N. kawakamii* and *N. otophora* was digested using an excess of enzyme (5 U μg^-1^ DNA, twice for 8 h). Digested DNAs were precipitated with isopropanol, washed with 70% ethanol and re-dissolved in TE to concentrations of about 150–200 ng μl^-1^.

For the emulsion PCR we essentially followed the protocol of Williams et al. [36]. Oil-surfactant mixture was prepared by thorough mixing of Span 80 (225 μl; SIGMA, USA), Tween 80 (20 μl; DIFCO, UK), Triton X-100 (2.5 μl; SIGMA, USA) and mineral oil (to 5 ml; SIGMA, USA) in a 15-ml centrifuge tube at 25°C. Four hundred μl of the oil-surfactant mixture was transferred into a CryoTube vial and stirred until use.

The composition of the aqueous phase (230 μl) for the emulsion was as follow: 1x PfuUltra II reaction buffer, 10 g l^-1^ BSA, 300 nM each forward and reverse primer, 200 μM each dNTP, 5 ng μl^-1^ of template DNA and 4.6 μl of PfuUltra II Fussion HS DNA Polymerase (Stratagene, USA). The amount of genomic DNA was reduced to a minimum in order to maintain an excess of aqueous droplets over template molecules. The tripartite structure of primers was as follows: 454 sequencing adaptors (italics), variable hexanucleotide TAG sequence (normal letter) and a gene specific 3’ end (bold). Primer A (5’-*CGTATCGCCTCCCTCGCGCCATCAG*TCGTAT**CTACACTGATGTATTCAACGAG**-3’) contained the conserved 18S gene; Primer B (5’-*CTATGCGCCTTGCCAGCCCGCTCAG*TCGTAT**CCGTTGCCGAGAGTCGTTT3**’) was derived from the 5.8S gene (Figure 1). The gene-specific parts are identical to 18S-FOR and 5.8S-REV2 primers used in previous projects [37] and do not seem to discriminate between individual alleles. Two hundred μl of the aqueous phase was added in a drop-wise manner to the 400 μl of stirred oil-surfactant mixture for over a period of 1.5-2.0 min. Stirring was continued for an additional 5 min and then the whitish emulsion was divided into 50 μl aliquots. Approximately 20 μl of aqueous phase was used as a control. The samples were overlaid with a drop of mineral oil and subjected to temperature-cycling: initial denaturation at 94°C for 3 min was followed by 25 cycles of 94°C for 30 s, 57°C for 30 s and 72°C for 40 s. The emulsified PCR reactions were pooled into a 1.5 ml microcentrifuge tube and centrifuged at 13,000g for 5 min at room temperature. The upper (oil) phase was removed. In order to break the emulsion, 2–3 extractions with water-saturated diethyl ether were performed (approximately 1 ml of ether was added, vortexed and the upper phase removed). Residual ether was removed by incubation 15 min at 42°C or by a centrifugation under vacuum for 5 min at room temperature. The PCR products were checked by agarose gel electrophoresis and cleaned using a PCR purification kit (Macherey-Nagel, Germany). The binding buffer was adjusted to remove all unincorporated primers according to the manufacturer’s recommendation.

### Amplicon sequencing

Samples containing 100–200 ng of each tagged PCR product were pooled and sent to EuroFins Operon MWG (Germany) for 454 sequencing. The pooled sample was sequenced from Primer A- and Primer B sides using a Roche GS FLX apparatus and Titanium series chemistry. Average length of reads was 350 to 450 base pairs and these reads are available at the NCBI Sequence Read Archive under the accession number SRA051350.

Amplicon variance analysis was carried out at Eurofins MWG Operon (Germany) and included Mira coverage mapping and syntheny plots. The SNPs were detected in the 454SNP/454HCDiff.files retrieved from the GsMapper. The criteria for clustering were as follows: All alignments were performed using the Smith-Waterman algorithm with default parameters with an identity threshold of e-100 and a maximum of 10 mutations per read. The reference sequences used for mapping were obtained from direct Sanger sequencing of 18S-5.8S PCR products. In general, half of the reads were successfully mapped to the reference sequences. The unmapped reads contained mostly shorter sequences or unrelated and highly variable sequences that were excluded from further analysis. The lengths of reference sequences were as follows: 313 bp of the 18S (all species), 240±2 bp of the ITS1. The 313 bp of 18S gene is delimited by positions 1689–1801 of the 18S gene from tomato (X51576.1); positions of ITS1 were according to the sequences deposited in GenBank (AJ492423.1, AJ492450.1, AJ492455.1, AJ492445.1, [27]). The sequences from major (>5% of total reads) clusters (equivalent to gene families) were imported to the BIOEDIT sequence editor [38] and checked by eye.

### Illumina genomic DNA sequencing

Sequence reads were obtained from Illumina sequencing of *N. sylvestris* (ac. ITB626) and *N. tomentosiformis* (ac. NIC 479/84) genomic DNA using the Illumina Genome Analyzer xII at the Genome Centre Queen Mary University of London, as described in Renny-Byfield et al. [23]. A random sample between 47-61% of the genome was sequenced for each species. Sequence reads are available at the SRA under the study accession number SRA045794.1. Illumina reads were mapped to ITS reference sequences and SNP and DIP (insertion/deletion) analysis was carried out using GLC Genomics Workbench with the following parameters/requirements: window length of 11 bp, maximum of 2 gaps, a minimum coverage of 4, variants should occur at a minimum frequency of 0.01, with a maximum of 1000 variants expected.

### Analysis of clones

ITS sequences were obtained by PCR amplification of genomic DNA. In a 25 μl reaction we used 0.1–1 ng genomic DNA as template, 500 nM each primer, 200 μM each dNTP, and 0.4 units of DyNAzyme II DNA polymerase (Finnzymes, Espoo, Finland). Cycling conditions were as follows: an initial denaturation step of 94°C for 180 s followed by 15–35 cycles of 94°C for 20 s, 57°C for 30 s and 72°C for 30 s with 18S-FOR (5’-GCGCTACACTGATGTATTCAACGAG-3’) and 5.8S-REV (5’-CGCAACTTGCGTTCAAAGACTCGA-3’) primers [37]. The products were purified using a PCR purification kit (Macherey-Nagel, Germany) and cloned into a dT vector (pDrive, Qiagen, Germany). Sequencing was carried out by dideoxy chain-termination at the Eurofins MWG Operon (Germany) using the SP6 and T7 primers. We considered the possibility of differential amplification of GC-rich and GC-poor templates [39,40] and included 5% dimethyl sulfoxide in the PCR reaction and found this had no effect on the proportion of amplified gene families.

### Southern blot hybridisation

DNA was extracted from fresh young leaves according to Kovarik et al. [41], digested with *Eco*RV restriction endonuclease (5 U μg^-1^ DNA, twice for 6 h), fractionated by gel electrophoresis and transferred to Hybond XL membranes (GE-Healthcare, UK) using alkaline capillary transfer. Membranes were hybridised with 32P-labelled DNA probe (DecaLabel DNA Labeling Kit, MBI Fermentas). Southern blot hybridisation was carried out in a 0.25 M sodium phosphate buffer (pH 7.0) supplemented with 7% (w/v) sodium dodecyl sulphate (SDS) at 65°C. Membranes were washed with 2x SSC, 0.1% SDS (twice for 5 min) and then with 0.2x SSC and 0.1% SDS (twice for 15 min at 65°C). The membranes were exposed to a Storage Phosphor Screen, scanned (Storm, GE-Healthcare, USA) and the signal was quantified using Image Quant (GE-Healthcare, USA). The DNA probe was an insert of the clone carrying the 18S gene sequence from tomato (GeneBank number X51576).

### Data access

All reads are available at the NCBI Sequence Read Archive (SRA) under the accession numbers SRA051350 and SRA045794.1. The cluster of reads used for mutation analysis can be accessed from: http://www.ibp.cz/local/data/matyasek/.

## Results

**Figure 2:**
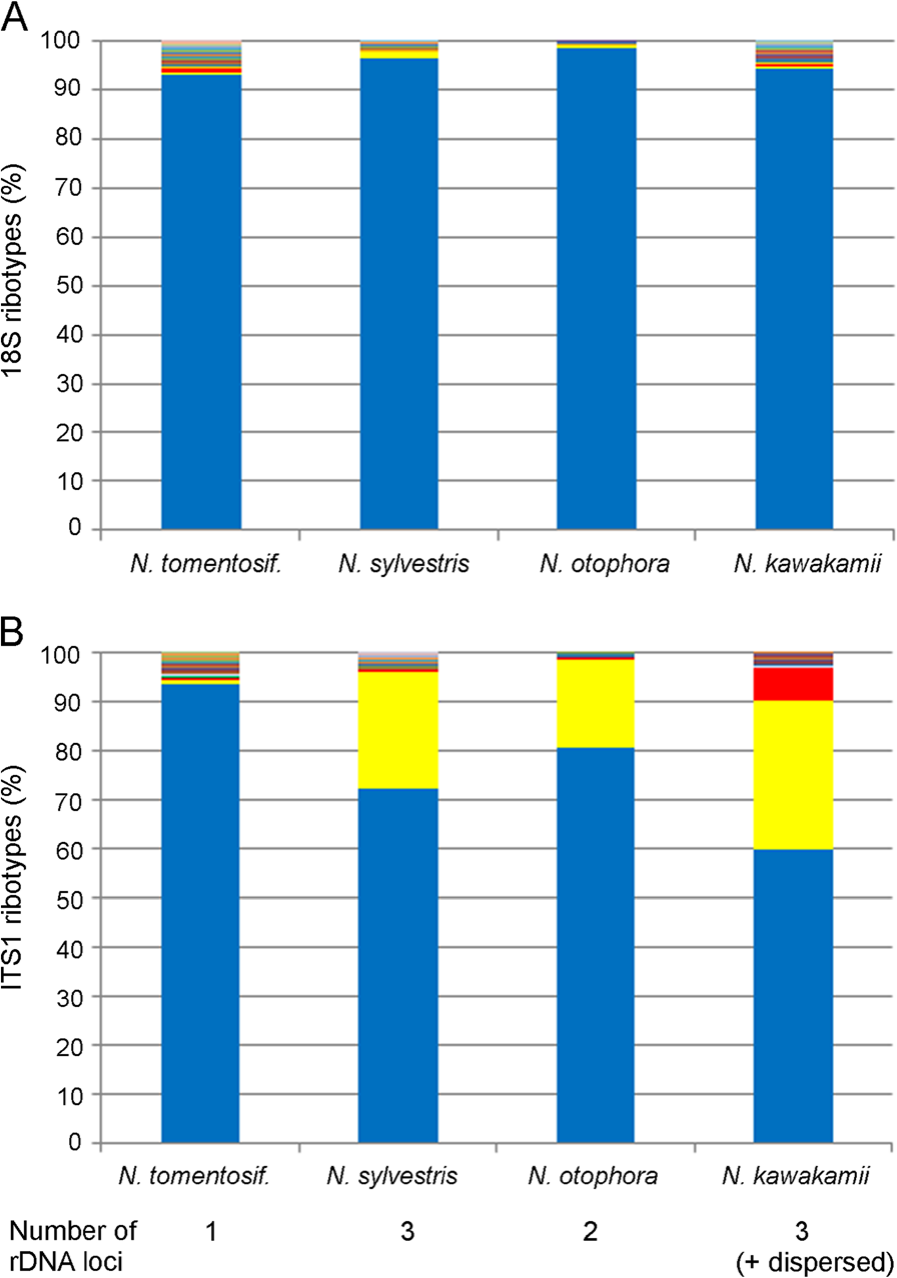
**Relative abundance of individual clusters in 454 generated (A) 18S rDNA and (B) ITS1 sequences.** The clusters are distinguished by SNPs, with clusters carrying indels having been merged with clusters that carry the same SNP profile.

We used emulsion PCR (for primer sites see Figure 1) to analyse the diversity of 18S and ITS1 regions of rDNA in four *Nicotiana* species, *N. tomentosiformis*, *N sylvestris*, *N. otophor*a and *N. kawakamii*. PCR amplicons were sequenced using Roche 454 pyrosequencing and the resulting reads were mapped to reference sequences obtained by direct Sanger sequencing of PCR products (Table 1). The aligned sequences were sorted into clusters, where each cluster contained one or more reads with unique sequence (Additional files 1, 2). We analysed the nature of SNPs that define clusters and found C➝T/G➝A transitions were the most frequent polymorphisms in both 18S and ITS1 sequences, likely to be a consequence of high frequency mutations caused by deamination of methyl cytosine to thymine (Additional file 3). The percentage contribution each cluster makes to the rDNA complement is shown in Additional file 4. In each species, there was a single cluster of 18S sequences that comprised more than 70% of the reads, while the remaining clusters were read-poor (less than 1% of the total reads). ITS1 sequences were more diverse, with two to six clusters exceeding 3% of reads depending on the species. However, sequencing using Illumina technology indicated that clusters defined by indels were likely to be artefacts (see later). Therefore we disregarded indels, and redistributed the reads to other clusters based on nucleotide polymorphisms only. Furthermore, singletons (clusters with one read) may also be sequencing and/or PCR artefacts, and so these were also excluded from further analysis. This markedly reduced the number of clusters (from >300 to less than 60) in each species (Figure 2). The majority (>90%) of 18S reads in each species occurred in one cluster, while the remaining clusters were read-poor (<1.5% of reads). Thus for each species 18S rDNA homogeneity is high, indicating that these regions are largely formed by one ribotype. In the case of ITS1 sequences, there are differences among species: in *N. tomentosiformis* there is a single abundant ITS1 cluster (ribotype); however, in *N. sylvestris*, *N. kawakamii* and *N. otophora* there are multiple read-rich ITS1 clusters (2–3 of which exceed 5% of the total number of reads).

**Table 1:** Summary of 454 sequencing data

**Species**	**Number of loci1**	**Copies/1C2**	**Reads mapped**		**Coverage3**		**Number of clusters**	
			**18S**	**ITS1**	**18S**	**ITS1**	**18S**	**ITS1**
*N. tomentosiformis*	1	1400	1190	1592	0.9	1.1	204	190
*N. sylvestris*	3	5100	2657	3882	0.5	0.8	339	483
*N. otophora*	2	1500	1469	2308	1.0	1.5	105	303
*N. kawakamii*	3+dispersed	2200	998	1093	0.5	0.5	138	213

^1^See Figure 6 and Plant rDNA database - http://www.plantrdnadatabase.com[42].

^2^The gene copies were initially determined by slot blot hybridisation [43] and later adjusted by calculation from the NGS data [22] using a http://w3lamc.umbr.cas.cz/profrep/ server. For *N. kawakamii* and *N. otophora* the copies were determined based on Southern blot signals (Figure 5).

^3^Mean read-depth: the number of aligned reads divided by rDNA copy number.

### Illumina sequencing

**Figure 3:**
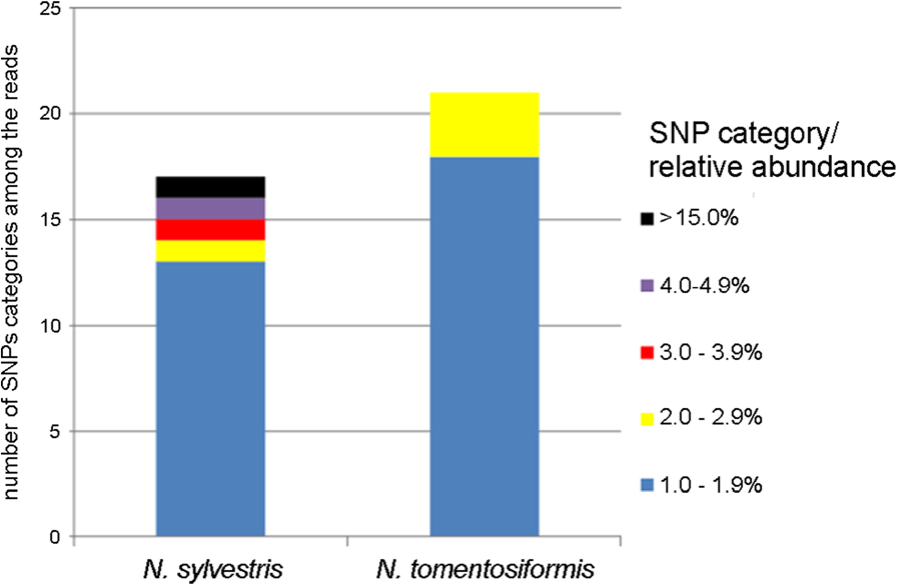
**Number of ITS1 variants occurring in each of five abundance categories for *N. sylvestris *or *N. tomentosiformis.*** Only SNPs that exceeded 1% of total Illumina reads in the appropriate species were considered. The graph reports the frequency of the most abundant SNPs – i.e. in *N. sylvestris* there is one major SNP that occurs in 15.5% of reads (black column) plus several minor SNPs occurring at lower relative abundance.

Illumina reads from *N. sylvestris* and *N. tomentosiformis* were mapped to reference ITS1 sequences obtained from Sanger sequencing of PCR products from both species using the “map reads to reference” function of CLC Genomics Workbench. The read-depth for each nucleotide position along the ITS region varied from 239 to 750. Subsequently, we analysed mutational variation using SNP and DIP detection algorithms in the CLC Genomics Workbench (Additional files 5, 6). Out of 240 nucleotide positions, there are 140 (58%) and 154 (64%) polymorphic sites in *N. tomentosiformis* and *N. sylvestris,* respectively. At variant positions, deviation from the most common nucleotide was rare, in most cases and did not exceed 1% of the reads, as previously observed in 454 sequence data. Substitution polymorphisms occurring at >1% frequency were counted and are presented graphically in Figure 3. Of the sites that are polymorphic, the frequency of the commonest SNP allele was high, with polymorphisms influencing only a minor fraction of sequences (minor SNPs). Of these minor SNPs, the commonest occurred in only 2.4% of reads in *N. tomentosiformis* and 15.5% of reads in *N. sylvestris* (Figure 3). The position of predominant polymorphic sites matched those identified by 454 amplicon sequencing.

### Comparing data between sequencing methods

To validate the results we compared frequencies of SNPs obtained with different sequencing methods across *N. sylvestris* ITS1 sequences (Additional file 7). We cloned 18S-ITS1 PCR products from *N. sylvestris* and sequenced (15 clones) by Sanger method. As expected, we did not observe any variation in the 18S region, confirming high levels of homogeneity. However, three clones (20%) contained a C➝T substitution at position 57 in ITS1. These clones were identical to the 454 reads in the second most abundant cluster (Figure 2, yellow field). Likewise SNP analysis of Illumina datasets revealed the same substitution, occurring at a frequency of 15.5% (Figure 3). Thus all three methods are broadly comparable.

Among the 3882 *N. sylvestris* ITS sequences derived from 454 data, there were 680 reads with indels and 800 reads harbouring substitutions (1.2 substitution/indel ratio). The ratio was significantly higher (18.1 substitution/indel ratio) among the Illumina reads indicating that most indels in the 454 data are likely to be artefacts. Indeed, visual inspection of sequences showed that most indels were actually located in homopolymeric tracts, where pyrosequencing is known to introduce errors [44]. Illumina seems to be less prone to indel artefacts, although even in this case, one abundant indel variant (15% reads) could not be traced among the 454 reads or Sanger sequences. We recommend caution when interpreting indel polymorphisms from such NGS data, and suggest using a combination of approaches.

### Phylogenetic relationships between the ITS1 families

**Figure 4:**
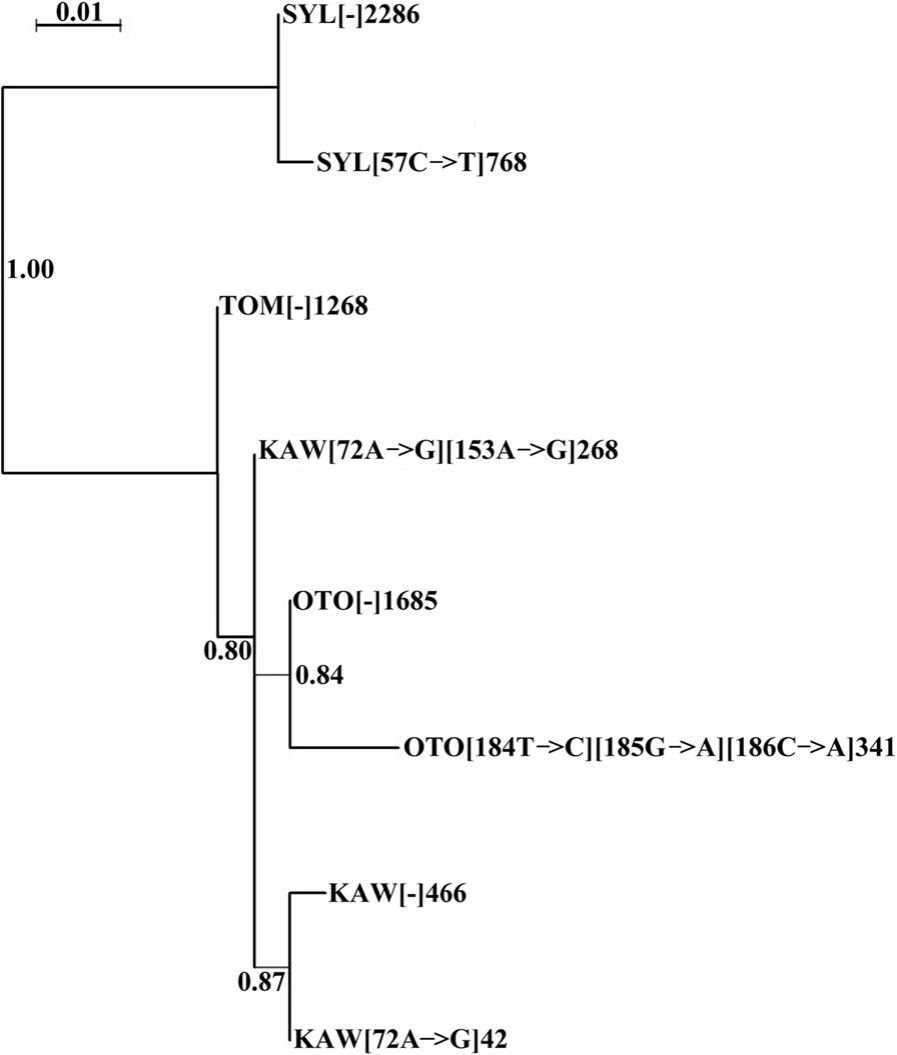
**Maximum likelihood tree showing relationships between major ITS families.** Sequences are from abundant clusters (>5% of reads) derived from Roche 454 sequencing. Branch support is indicated at each node and was calculated using 500 ML replicates. SYL = *N. sylvestris*, KAW = *N. kawakamii*, TOM = *N. tomentosiformis*, OTO = *N. otophora*. Mutations giving rise to each ribotype are listed and shown in brackets after the species abbreviation. The last number indicates the number of reads in the cluster. Scale indicates the base substitutions per site.

To determine phylogenetic relationships between ITS ribotypes, we extracted a single ITS ribotype from *N. tomentosiformis*, two from *N. sylvestris* and *N. otophora*, and three from *N. kawakamii*, based on a minimum 5% representation in the corresponding species. The sequences were aligned and phylogenetic trees constructed. Maximum Likelihood (ML) and Neighbour joining trees had similar topologies. The ML tree (Figure 4) revealed a relatively large phylogenetic distance between ITS1 ribotype in *N. sylvestris* and ribotypes in the three other species. This is expected since *N. sylvestris* is placed within the section *Sylvestres* while the rest of species belong to section *Tomentosae*[45]. Amongst sequences from section *Tomentosae*, the two ITS1 families of *N. otophora* form a distinct clade, whilst those from *N. kawakamii* fall into two groups. Next, using BLAST searches we determined if ITS variants in one species were present in low copy number in other species. However, abundant ribotypes appeared to be unique for each species and did not appear in other species, even among low-copy clusters.

### Southern blot hybridisation

**Figure 5:**
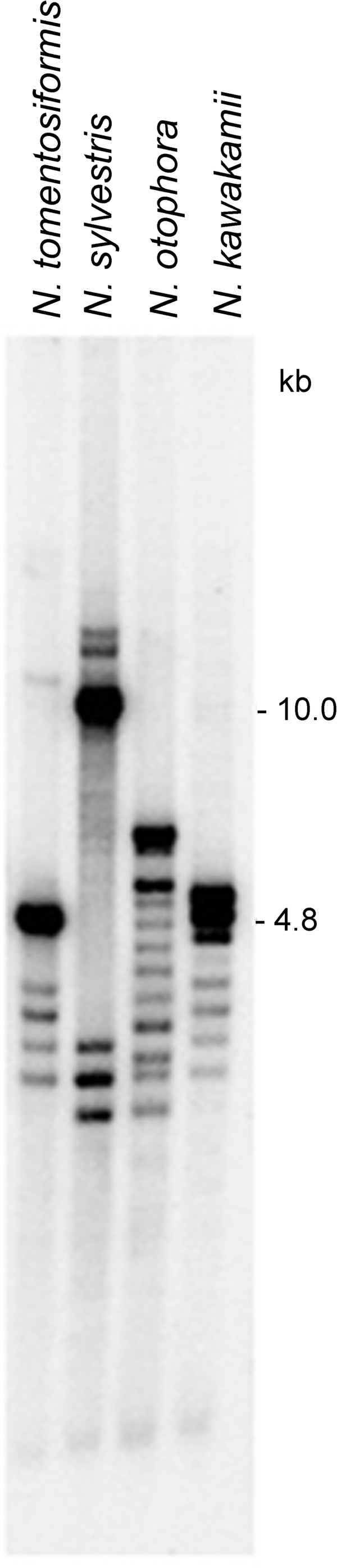
**Southern blot hybridisation shows the presence of multiple IGS variants in the *Nicotiana *genomes.** There were at least 14 *Eco*RV bands in *N. otophora.*

The intergenic spacer (IGS, Figure 1) is internally repetitive making the mutation analysis of this region difficult. The individual sub-repeats within the IGS showed differential coverage in the 454 NGS data sets (Additional file 8). We therefore carried out Southern blot hybridisation to determine intragenomic homogeneity of IGS sequences. Genomic DNAs were digested with EcoRV restriction enzyme to release part of the IGS and a complete 18S gene. The restriction fragments were probed with 18S rDNA (Figure 5). It is evident that besides a major single hybridisation band there were additional bands with a weaker hybridisation signal. The fainter fragments formed a ladder of bands spaced at 135 bp intervals, indicating point mutations and/or variable number of A1/A2 subrepeats located downstream of the promoter [30,34]. Thus, as with the IGS displays significant levels of intragenomic heterogeneity. Analysis of sequence coverage revealed substantial differences in the lengths and homogeneity of IGS subrepeats between *N. tomentosiformis* and *N. sylvestris* (Additional file 8). The upstream and downstream repeated elements have differentially amplified between both species [34].

## Discussion

To study concerted evolution of rDNA arrays during the divergence of diploid *Nicotiana* species, we analysed SNPs in the 18S (coding) and ITS1 (non-coding) regions using Roche 454 and Illumina technology. The coverage of both 18S (3’ end) and ITS1 regions of rDNA was close to 1, meaning that most rDNA variants are likely to be represented in our dataset. We show that 18S sequences are more homogenous than ITS1; a pattern that could arise from incomplete rounds of homogenisation and stronger purifying selection acting on the 18S genic region. By analysing genomic and cytogenetic data, we find evidence that, in *Nicotiana*, the separation of rDNA arrays between chromosomes influences patterns of rDNA homogenisation. We explore these ideas in more detail below.

### Homogeneity in coding and non-coding regions of the rDNA unit

*Nicotiana tomentosiformis* has only one read rich-cluster (defined as a cluster comprising more than 5% of the reads) for both 18S and ITS1 sequences (Figure 2). These data demonstrate near complete homogenisation of both 18S and ITS1 sequences to a single ribotype. For the other diploid species *N. sylvestris*, *N. kawakamii* and *N. otophora* there was also a single read rich 18S rDNA cluster, but there were two or three read rich ITS1 clusters that differed by 1–3 substitutions per 240 bp (0.4-1.3% divergence; Figure 2). Thus, within each of these species the 18S gene is more homogenous than the ITS1. The average number of major ITS1 variants in *Nicotiana* is similar to the average number (2–3) of ITS2 ribotypes recently determined for a wide range of plant species by pyrosequencing [14] corroborating the hypothesis that amplification and fixation of rDNA variants could be larger in plants than in yeasts [46] and *Drosophila*[47]. Consistent with previous studies [48,49], for IGS sequences there is even greater complexity in the *Nicotiana* species analysed (Figure 5) probably due to its sub-repeated nature ([34] and Additional file 8). Increasing genetic variation, 18S < ITS1 < IGS likely reflect reducing strengths of selection. Probably the IGS homogenises by similar mechanisms (e.g. unequal cross over) as the rest of the unit since the first and last A1/A2-subrepeats (Figure 1) are more variable than those of the central region [50], consistent with distance-dependent models of repeat evolution [51,52].

### Most rDNA ribotypes have not survived species divergence in *Nicotiana*

**Figure 6:**
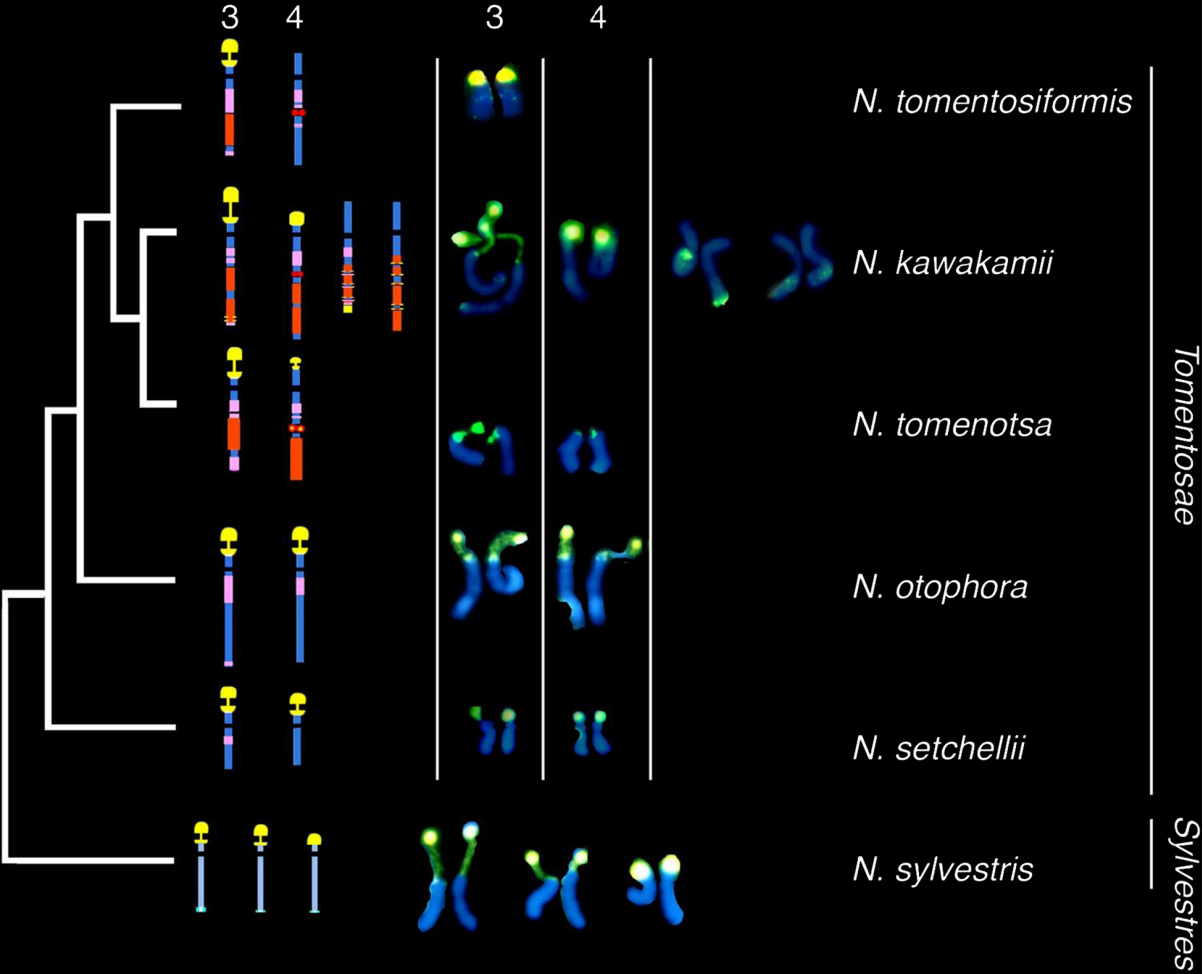
**Phylogenetic relationships between species in section *Tomentosae *and *N. sylvestris *as determined by the distribution of repetitive DNA markers.** 35S rDNA – yellow; tandem repeats: GRS –pink, NTRS - orange, GRD3 - red, GRD5 - red with yellow centre, HRS60 - blue and white (found only in *N. sylvestris*). Fluorescence *in situ* hybridisation of 35S rDNA on *Nicotiana* chromosomes (yellow signals) with chromosomes counterstained blue with DAPI. Note, dispersed yellow signals, particularly on the fourth pair of *N. kawakamii* chromosomes. Data and interpretative diagrams are taken from [17].

Analyses of the occurrence and distribution of repetitive DNA sequences [17,53] and phylogenetic analyses of plastid (trnL-intron, trnL-F, trnS-G, ndhF, matK; [54]) and glutamine synthase [25] sequences indicate that *N. tomentosiformis* is recently derived within the section. Fluorescence *in situ* hybridisation studies indicate that the ancestral organisation for the section is most likely to have been two 35S rDNA loci, located on chromosomes 3 and 4 (Figure 6, the homeology is supported by cytogenetic observations of satellite and endogenous virus repeats [17,55]). If so, there has been a gain in locus number in the lineage leading to *N. kawakamii* and loss of a locus in the lineage leading to *N. tomentosiformis*[17]. *Nicotiana sylvestris* occurs in the distantly related section *Sylvestres* and has three 35S rDNA loci, the homeologous relationship to loci with section *Tomentosae* is currently unknown. Despite the more derived position of *N. tomentosiformis* in *Tomentosae*, the one major ribotype identified is more similar to the *N. sylvestris* ribotype than others in section *Tomentosae*, indicative of array homogenisation maintaining an ancestral-like unit. For *N. otophora* and *N. sylvestris*, the ribotypes fall into species-specific clades. For *N. kawakamii*, two copy types form a clade and the third copy an independent clade (Figure 4), revealing the amplification of two distinct variants in this species. It is unlikely that these data are explained by incomplete lineage sorting [10,56,57] of mixed rDNA arrays, since the ‘major’ ITS ribotypes in each species was not found in any other species. Indeed, there is evidence for the rapid establishment of ribotypes in other species: for example in the genus *Hordeum*, it is estimated that complete ITS replacement occurs within ~3.5 myrs [58] and deep sequencing of *Arabidopsis thaliana* show that ITS variants are differentially amplified between ecotypes [59]. Furthermore, in *Nicotiana* polyploids, we have estimated that there is near complete replacement of rDNA repeats in less than one million years [22,35]. Thus the rate of ribotype homogenisation appears to exceed the rate of speciation.

### Chromosomal locus separation as a driver of ITS complexity

Large scale analysis of rDNA repeats in yeasts [46] and *Drosophila*[47] has also revealed differences among rDNA units of the same species. However, unlike the pattern we report here, intragenomic homogeneity is similar in coding and non-coding regions of each species, although slightly higher variation was observed in *Drosophila,* where rDNA sequences are located at several chromosomal loci. In explaining the level of variation, it is suggestive that the Rosaceae [60,61] and Cactaceae [9], in which large numbers of ITS variants were observed, also have a high (>5) number of rDNA loci [62,63]. A comparable pattern is reported within the grasshopper species *Podisma pedestris*, in which some populations have a larger number of rDNA loci and a greater complexity of rDNA units [64]. A similar correlation between locus number (Figure 6) and ITS1 complexity (Figure 2) emerges from our results. For example, *N. tomentosiformis*, with a single locus, has highly homogeneous 18S and ITS1 sequences, resembling the situation in unicellular yeast. On the other hand, *N. kawakamii* with four loci had the highest variability amongst ITS sequences.

**Figure 7:**
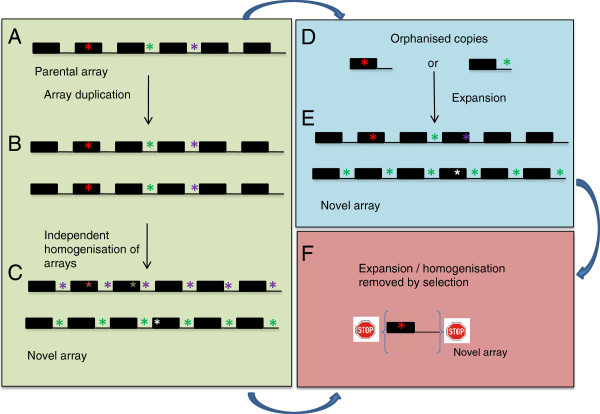
**A theoretical model to explain rDNA divergence.** Ribosomal DNA arrays (black box) separated by spacer sequences (black line) with some units carrying mutations (asterisks). Green panel: (**A**) 35S rDNA locus comprising rDNA arrays with a few mutations. (**B**) Duplication of the 35S rDNA locus, followed by (**C**) more rapid intrachromosomal homogenisation than interchromosomal homogenisation, leading to genetic divergence of both arrays and an increase in rDNA complexity. Mechanism would involve unequal recombination and gene conversion [2,69]. Blue panel: (**D**) Orphaned 35S rDNA copies may (**E**) nucleate locus expansion, resulting in long arrays of homogenous sequences. Pink panel: (**F**) Amplification of non-functional units will be selected against and lost from the population partially corroborating birth-and-death model of rDNA evolution [7,8].

Why then is there a generally greater number of ribotype variants (Table 1) in species with more rDNA loci? The relationship could be explained if the rates of intrachromosomal homogenisation exceed those of interchromosomal homogenisation as has been proposed to explain the divergence of rDNA [65], satellite DNA [66] and retroelements [67]. The dynamics of homogenisation are analogous to the effects of gene flow: just as reduced gene flow allows variation to accumulate between geographically-separated populations, low levels of homogenisation between different chromosomal loci would allow the accumulation of different ribotypes (see Figure 7B-C). Another explanation is analogous to the effects of founder events in classical population genetics: if new rDNA loci are established by the movement or copying of a small number of units to a new location followed by an expansion in the number of these units (Figure 7D-E), then that too would result in higher inter-chromosomal variation. The patterns would also be shaped by selection: amplified rDNA variants containing deleterious mutations would be selected against (Figure 7E-F), partially explaining why we did not observe variation in the coding region (Figure 2). In contrast, functional arrays may rapidly expand to saturate the rDNA population, reducing selection pressures on older, more degenerate arrays. Certainly rDNA units incapacitated by transposon insertions amplify to considerable extent in *Drosophila*[68], suggesting differences in the levels to which individual genomes tolerate non-functional copies.

The rDNA variants could have arisen either through the amplification of low copy number units (Figure 7D-E), or through locus duplication (Figure 7A-C). Although the latter possibility seems to be a more likely there appears to be sufficient evolutionary time for homogenisation of a large number of units from a single orphaned copy (Figure 7D): theoretical computer simulations imply that the time taken for sequences arranged in tandem to homogenise is a quadratic function of their copy number [5]. It follows that a typical array comprising about 1000 units would homogenise within ~10^6^ years. The age of *Nicotiana* diploid species (~10^7^ years, [27]) exceeds that limit; the period since the speciation split of *Nicotiana* would therefore appear sufficiently long for the replacement of thousands of rDNA units in the genomes, which would explain why we do not detect common ribotypes shared between species (inherited from the progenitors), even in low frequency clusters. There is some indirect experimental support for the hypothesis that new loci may arise by the amplification of orphanised or low copy number rDNA sequences:

1. Low copy numbers of rDNA units (or parts of the unit) can be found dispersed across the *Arabidopsis*[70] and some animal [71,72] genomes. IGS dispersion may not be uncommon in plant genomes since it has also been reported in other species, and proposed to be one of the mechanisms leading to formation of novel high copy satellites [73-75]. Indeed, we have observed this phenomenon in *Nicotiana* section *Tomentosae*, where there is dispersion of IGS sequences and 35S rDNA across the genome [30]. Such units could potentially nucleate the expansion of active rDNA loci. Interestingly, *N. kawakamii*, with the highest level of rDNA dispersion (Figure 6), also has the most heterogeneous ITS1 ribotypes.

2. New loci and units have been shown to evolve rapidly in early generations of synthetic allopolyploids [31,76-79] or in response to DNA damage [80]. In an allotetraploid line of tobacco, we observed amplification of new IGS types at a new rDNA locus [30].

3. Transposable elements may be vehicles for rDNA mobility and the generation of orphaned rDNA units across the genome. For example in wheat mobile rDNA sites may be connected with meiotic activity of En/Spm elements [81], while Ty1/Copia elements have been found in the IGS of *Allium cernuum*[82].

## Conclusions

The results here indicate that ITS1 is more heterogeneous than 18S, particularly when more than one rDNA locus is present. Low homogenisation frequency between rDNA arrays at different loci, together with strong selection pressures imposed on coding regions, would allow co-existence of repeats with identical coding but more variable non-coding regions. We suggest that locus duplication or amplification of orphaned rDNA units can be vehicles for rDNA divergence (Figure 7).

## Competing interests

The authors declare that they have no competing interests.

## Authors' contributions

AK, ARL and RN designed the study. AK, ARL, SRB and RN wrote the paper. RM, JF and MAG carried out most of the molecular biology part of the work. SRB, JM and RN carried out bioinformatic studies. All authors read and approved the final manuscript.

## Supplementary Material

Additional file 1 **List of 18S gene clusters from 454 amplicon sequencing.** Description: Each MsExcell list contains information about cluster ID, mutation pattern, number of reads, number of reads expressed as a percentage and total number of reads for a given species. For *N*. *sylvestris* and *N. tomentosiformis,* positions of variable sites read the non-coding DNA strands; for *N. otophora* and *N. kawakamii,* these read the coding strands. Click here for file

Additional file 2 **List of ITS1 clusters from 454 amplicon sequencing.** The MsExcell charts are organized as in Additional file 1. Click here for file

Additional file 3 Substitution mutation patterns in ITS1 region (single read clusters were excluded). Click here for file

Additional file 4 **Abundance of individual clusters (number of reads/total number of reads per cluster, expressed as a percentage) in 454 sequencing data from *N. tomentosiformis, N. sylvestris, N. otophora *and *N. kawakamii *ITS1 and 18S rDNA sequences.** The clusters are distinguished by polymorphisms, i.e. SNPs or indels. Click here for file

Additional file 5 **Output of SNP algorithm in CLC genomics of Illumina ITS1 reads from *N. sylvestris *and *N. tomentosiformis.*** Click here for file

Additional file 6 **Output of DIP algorithm in CLC genomics of Illumina ITS1 reads from *N. sylvestris *and *N. tomentosiformis.*** Click here for file

Additional file 7 **Comparison of polymorphisms analyzed by different sequencing methods.** The data are for *N. sylvestris* ITS1. Position (coding DNA strand) and type of mutation is in brackets : [−] – no mutation (usually the most abundant cluster), [57:C>T] – substitution C into T; [49:50>G] – insertion of G between nucleotides 49 and 50. Click here for file

Additional file 8 **Representation of IGS sequences in 454 reads.** The plots show number of similarity hits along (**A**) *N. sylvestris* and (**B**) *N. tomentosiformis*, obtained in BLASTN searches against 454 reads from *N. sylvestris* and *N. tomentosiformis*, respectively [22]. The analysis was run using the PROFREP server (http://w3lamc.umbr.cas.cz/profrep/public/) with e-value cutoff of 1e-15. The curves were smoothed by value averaging in a 10-bp sliding window. Conversion of the hit numbers to genomic copy numbers (per 1C) based on genome coverage of the 454 sequencing is provided on the right side of the plots. Click here for file
