# Supplementary material for: Next generation sequencing analysis reveals a relationship between rDNA unit diversity and locus number in Nicotiana diploids
Source: BMC Genomics. 2012 Dec 23;13:722. doi: 10.1186/1471-2164-13-722 (PMC3563450; doi:10.1186/1471-2164-13-722)
Supplement: Additional file 3 — Substitution mutation patterns in ITS1 region (single read clusters were excluded). [file 1471-2164-13-722-S3.pptx]

## Slide 1
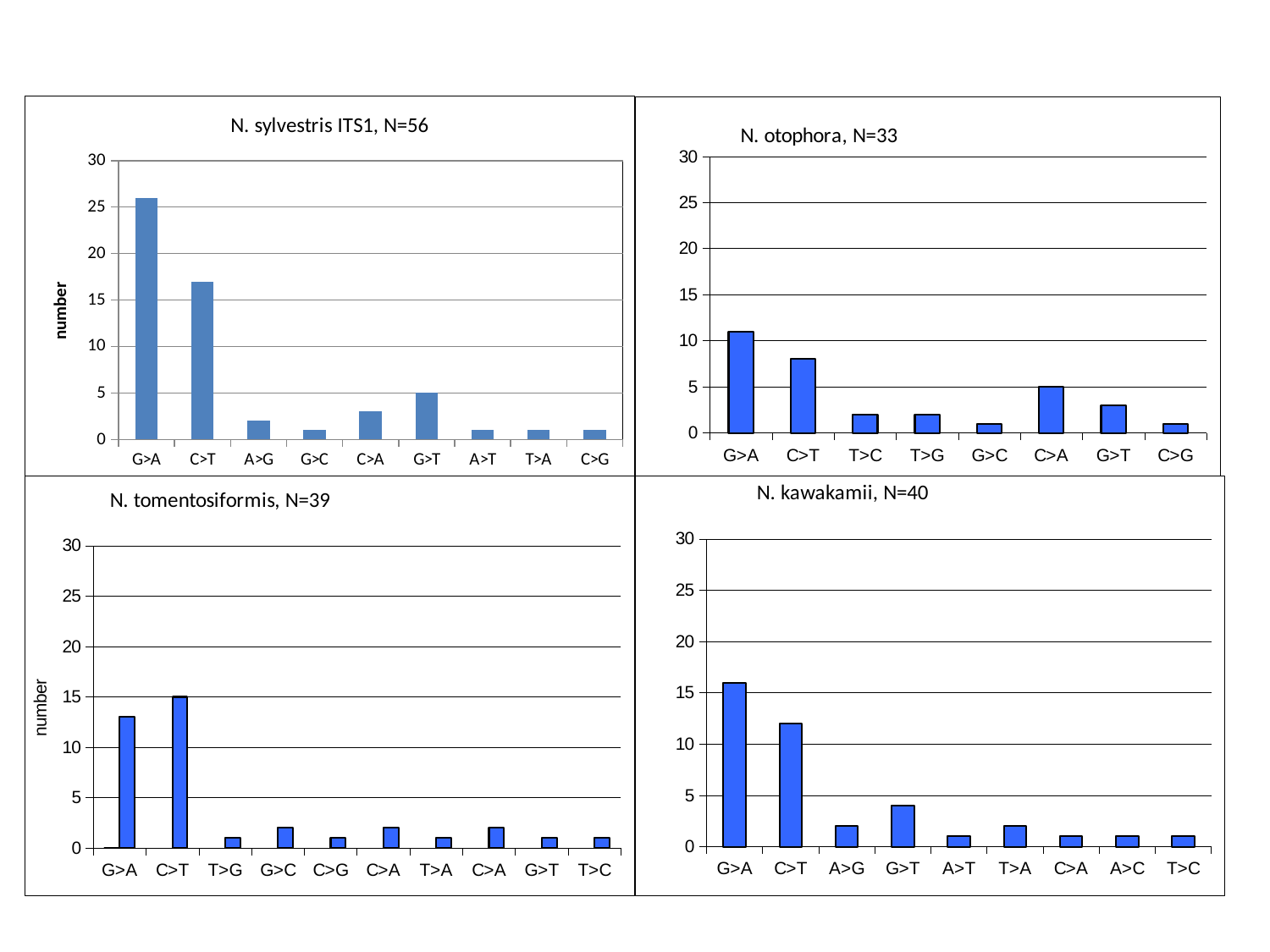

### Chart: N. sylvestris ITS1, N=56
| Category | |
|---|---|
| G>A | 26.0 |
| C>T | 17.0 |
| A>G | 2.0 |
| G>C | 1.0 |
| C>A | 3.0 |
| G>T | 5.0 |
| A>T | 1.0 |
| T>A | 1.0 |
| C>G | 1.0 |
### Chart: N. otophora, N=33
| Category | G>A C>T T>C T>G G>C C>A G>T |
|---|---|
| G>A | 11.0 |
| C>T | 8.0 |
| T>C | 2.0 |
| T>G | 2.0 |
| G>C | 1.0 |
| C>A | 5.0 |
| G>T | 3.0 |
| C>G | 1.0 |
[unsupported chart]
### Chart: N. kawakamii, N=40
| Category | G>A C>T A>G G>T A>T T>A C>A A>C |
|---|---|
| G>A | 16.0 |
| C>T | 12.0 |
| A>G | 2.0 |
| G>T | 4.0 |
| A>T | 1.0 |
| T>A | 2.0 |
| C>A | 1.0 |
| A>C | 1.0 |
| T>C | 1.0 |
