# Supplementary figures and images for: Next generation sequencing analysis reveals a relationship between rDNA unit diversity and locus number in Nicotiana diploids
Source: BMC Genomics. 2012 Dec 23;13:722. doi: 10.1186/1471-2164-13-722 (PMC3563450; doi:10.1186/1471-2164-13-722)

## Slide 1
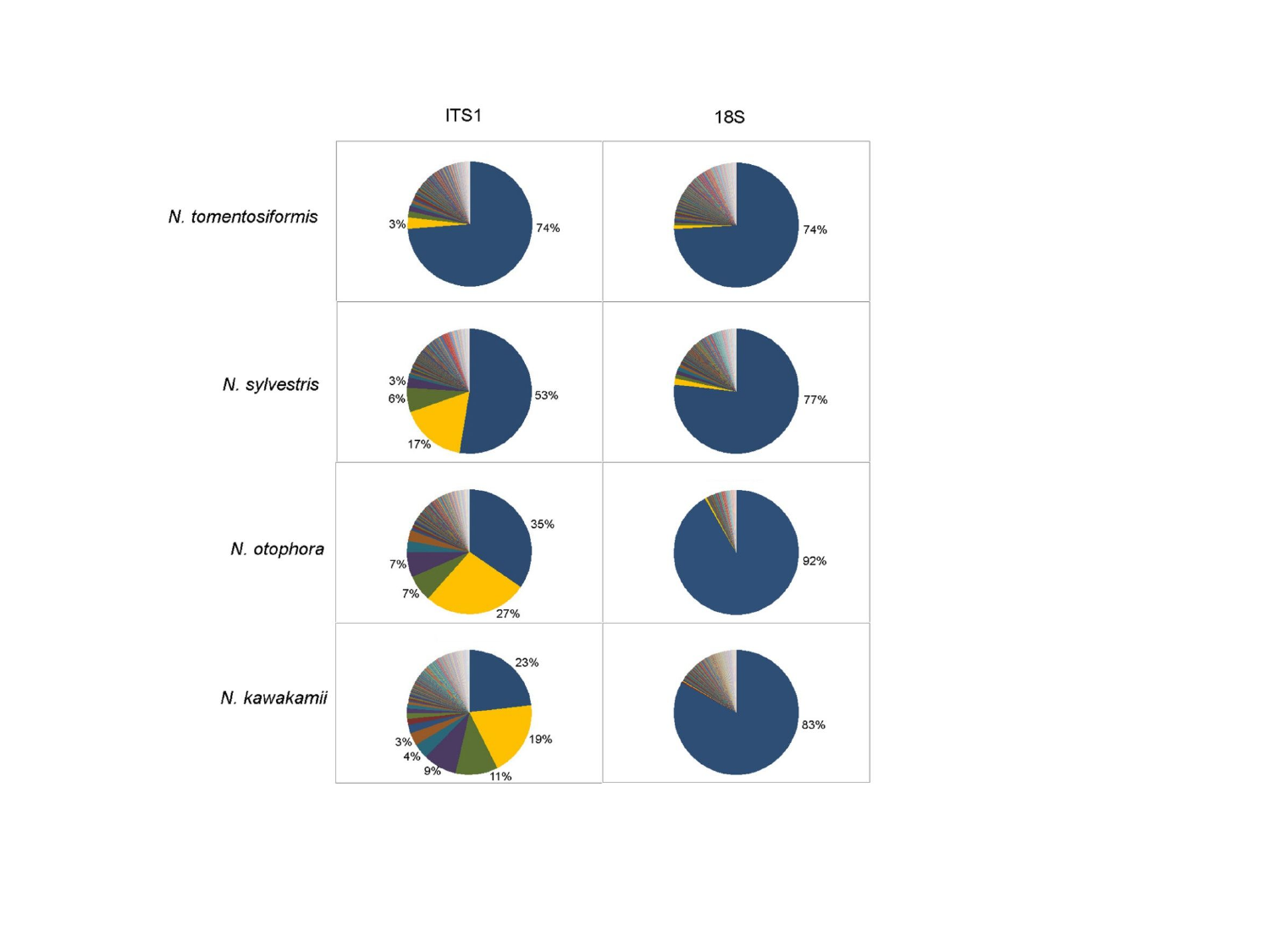

Supplement: Additional file 4 — Abundance of individual clusters (number of reads/total number of reads per cluster, expressed as a percentage) in 454 sequencing data from N. tomentosiformis, N. sylvestris, N. otophora and N. kawakamii ITS1 and 18S rDNA sequences. The clusters are distinguished by polymorphisms, i.e. SNPs or indels. [file 1471-2164-13-722-S4.pptx]

## Slide 1
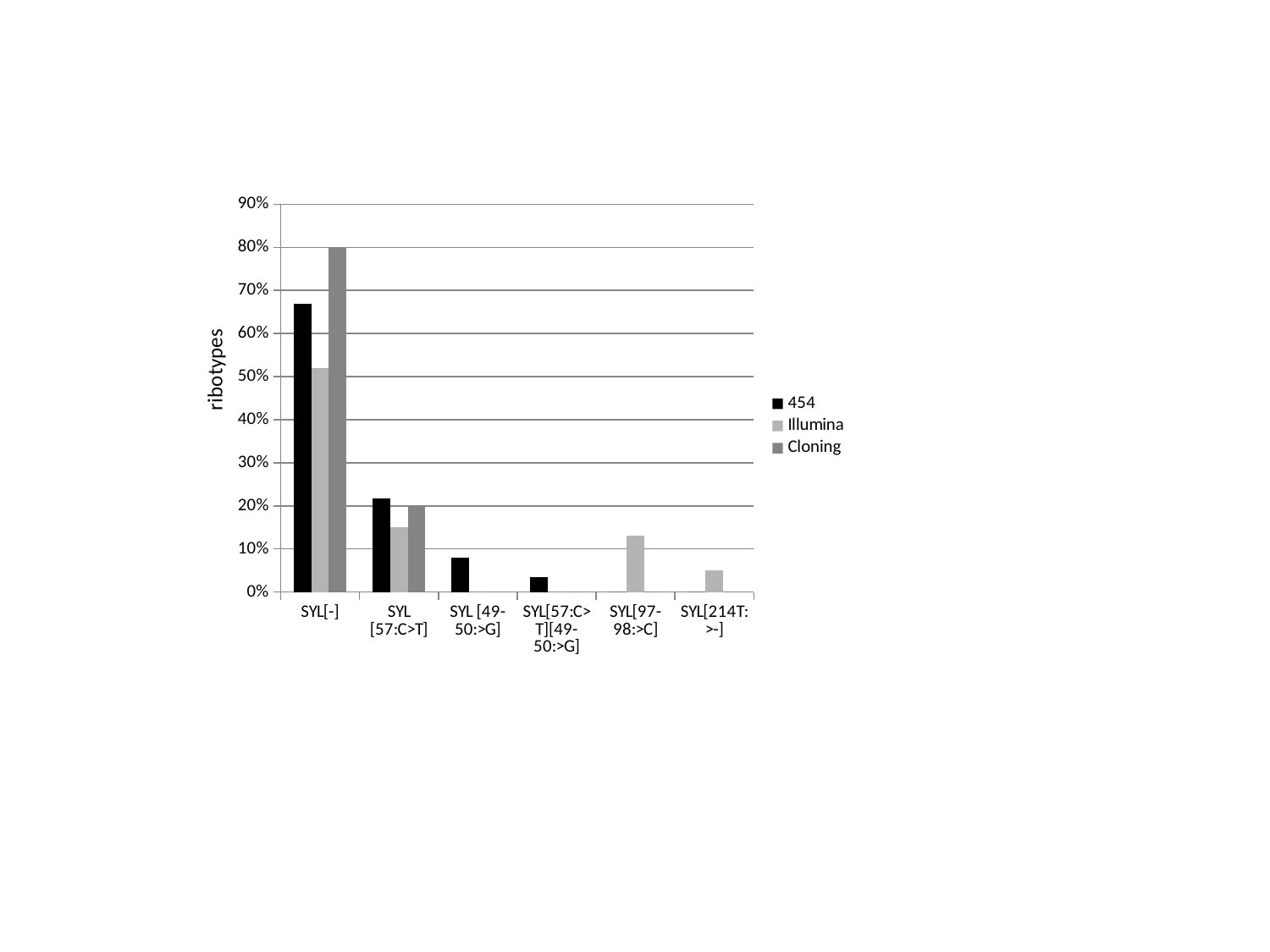

### Chart
| Category | 454 | Illumina | Cloning |
|---|---|---|---|
| SYL[-] | 0.6683038637851998 | 0.52 | 0.8 |
| SYL [57:C>T] | 0.21676489849377864 | 0.15 | 0.2 |
| SYL [49-50:>G] | 0.0802226588081205 | 0.0 | 0.0 |
| SYL[57:C>T][49-50:>G] | 0.03470857891290111 | 0.0 | 0.0 |
| SYL[97-98:>C] | 0.0 | 0.13 | 0.0 |
| SYL[214T:>-] | 0.0 | 0.05 | 0.0 |ribotypes

Supplement: Additional file 7 — Comparison of polymorphisms analyzed by different sequencing methods. The data are for N. sylvestris ITS1. Position (coding DNA strand) and type of mutation is in brackets : [−] – no mutation (usually the most abundant cluster), [57:C>T] – substitution C into T; [49:50>G] – insertion of G between nucleotides 49 and 50. [file 1471-2164-13-722-S7.pptx]

## Slide 1
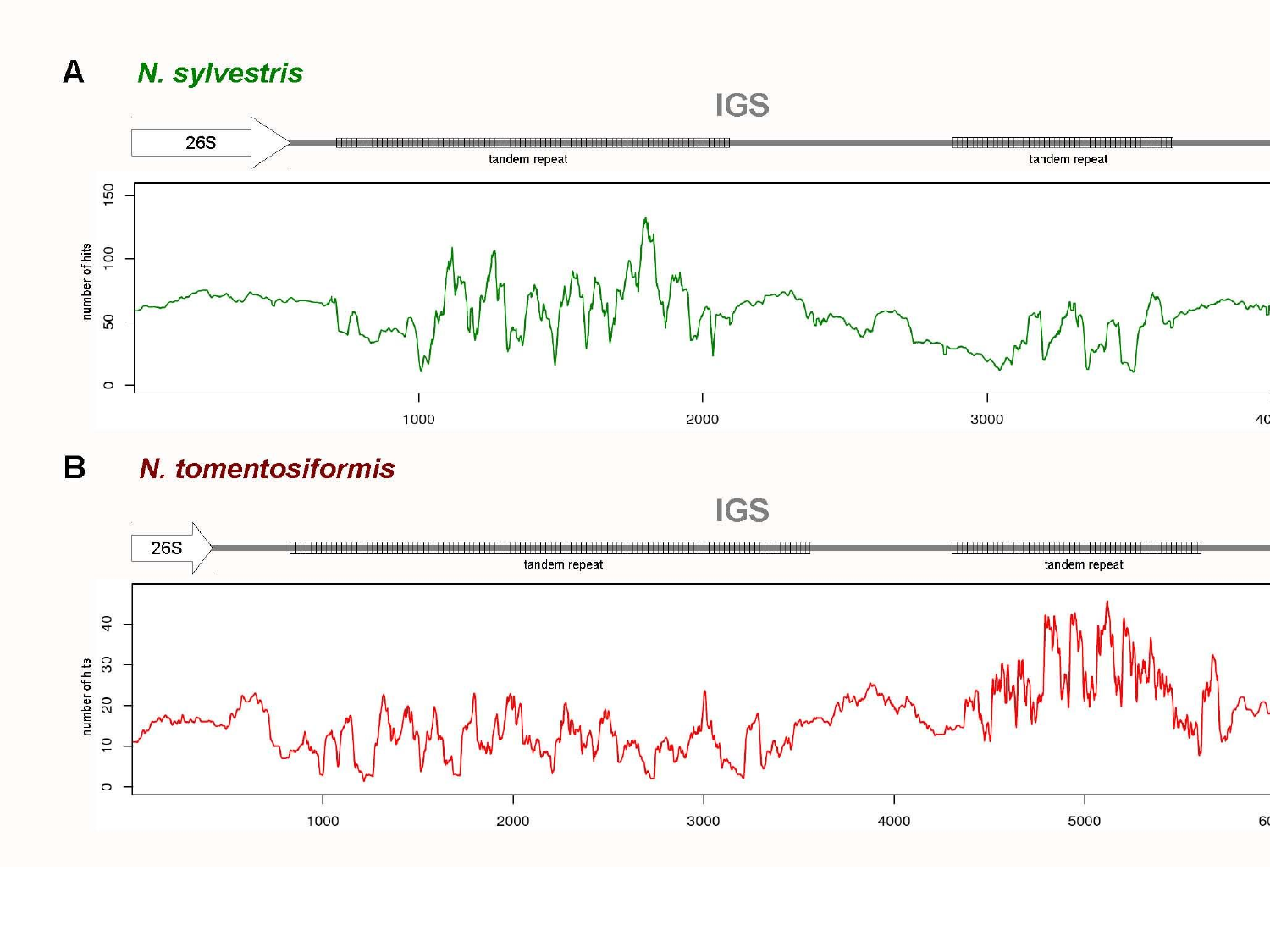

#

Supplement: Additional file 8 — Representation of IGS sequences in 454 reads. The plots show number of similarity hits along (A) N. sylvestris and (B) N. tomentosiformis, obtained in BLASTN searches against 454 reads from N. sylvestris and N. tomentosiformis, respectively [22]. The analysis was run using the PROFREP server (http://w3lamc.umbr.cas.cz/profrep/public/) with e-value cutoff of 1e-15. The curves were smoothed by value averaging in a 10-bp sliding window. Conversion of the hit numbers to genomic copy numbers (per 1C) based on genome coverage of the 454 sequencing is provided on the right side of the plots. [file 1471-2164-13-722-S8.pptx]
